# Investıgatıon of the effects of treatment with enoxaparın sodıum and hyperbarıc oxygen therapy on the recovery of rats wıth achılles tendon rupture

**DOI:** 10.1186/s12891-024-07694-6

**Published:** 2024-07-18

**Authors:** Cafer Erman Aytekin, Yalcın Turhan, Zekeriya Okan Karaduman, Mehmet Arıcan, Sönmez Saglam, Sinem Kantarcıoglu Coskun, Veysel Uludag

**Affiliations:** 1 Faculty of Medicine, Department of Orthopaedics and Traumatology, Duzce University, Duzce, Türkiye; 2 Faculty of Medicine, Department of Pathology, Duzce University, Duzce, Türkiye

**Keywords:** Achilles tendon rupture, Hyperbaric oxygen therapy, Enoxaparin sodium, Rat

## Abstract

**Purpose:**

In this study, we aimed to investigate the effects of hyperbaric oxygen therapy and enoxaparin sodium, which are known to accelerate bone tissue healing as well as tendon and soft tissue healing, on the healing of Achilles tendon rupture.

**Methods:**

Thirty-six rats were used in the present study. All rats were divided into groups of nine. The groups were the enoxaparin sodium group, enoxaparin sodium and hyperbaric oxygen group, hyperbaric oxygen group and control group. After 21 days, the process was completed, and the rats were sacrificed. Achilles tendon samples were evaluated histopathologically.

**Results:**

The groups were compared according to the results of statistical analysis based on the histopathological data. There was no significant difference between the groups in terms of acute inflammation (*p* = 0.785) or chronic inflammation (*p* = 0.827) scores, but there were significant differences in neovascularization (*p* = 0.009), proliferation (*p* < 0.001) and fibrosis (*p* = 0.006) scores.

**Conclusion:**

Our study showed that the use of enoxaparin sodium and hyperbaric oxygen had a positive effect on the healing of the Achilles tendon. Based on these results, we believe that the use of enoxaparin sodium and hyperbaric oxygen therapy after Achilles tendon rupture will be beneficial for healing and preventing complications.

## Background

Although the Achilles tendon is one of the strongest tendons in the human body, rupture rates have increased significantly in recent years with increasing physical activity in middle-aged and older people. There have been many studies on the treatment and healing of Achilles tendon rupture, and research on tendon healing is still ongoing to accelerate the return to social life after rupture and to reduce rerupture rates.

The treatment of tendon pathologies, which are becoming increasingly common as a result of prolonged life expectancy and the increasing involvement of sporting activities for hobbies or health purposes, is maintaining importance in orthopedic surgery [1]. Although significant gains have been achieved with numerous studies and many surgical methods developed in this field, the difficulties and complications encountered continue to play an important role in the treatment process [2–4]. Tendons are the most important parts of the musculoskeletal system and transfer the energy produced by the muscles to the bone. Because of the functions of these devices, tendon pathologies cause significant morbidity. Tendon injuries may occur due to acute trauma, chronic inflammatory conditions or overuse [5]. Tendon healing is a slow, progressive process [6]. Because of these problems, new studies are constantly being conducted to improve treatment.

HBO therapy is a treatment method applied for many diseases; patients intermittently inhale 100% oxygen via various methods (mask, endotracheal tube, etc.) under pressure greater than one atmosphere in a closed pressure chamber [7]. The efficacy of HBO therapy, which has been shown to have many positive effects on tissues, is due to hyperoxygenation, neovascularization, antimicrobial activity, pressure effects, vasoconstriction and a reduction in reperfusion damage [8]. There are studies with successful results in many pathological conditions, such as necrotizing soft tissue infections, acute traumatic ischemia, crush injury, compartment syndrome, problematic wounds, problematic skin grafts and flaps, refractory osteomyelitis, osteonecrosis, sports injuries, fracture healing, nerve healing, carbon monoxide poisoning, and decompression sickness; additionally, there are cases where HBO therapy is added to treatment modalities.

Antithrombotic agents are now routinely used for thromboembolism prophylaxis in thromboembolism-prone patients before and after surgery in patients with major orthopedic injuries. Several studies have reported that low-molecular-weight heparin (LMWH) positively affects wound healing, increases macrophage stimulation, increases new vessel formation and decreases fibrous tissue formation [9, 10]. LMWH were developed to overcome the disadvantages of intravenous standard heparin. These patients do not require monitoring due to their predictable activity and do not require heparin. Moreover, the risk of thrombocytopenia is lower. Physiologically, LMWH shows high anti-Factor Xa activation [11] but relatively low anti-Factor IIa activity. This has made it an inevitable pharmacological agent for reducing deep vein thrombosis (DVT) risk, especially after orthopedic surgeries [12]. The American Academy of Orthopaedic Surgeons recommends LMWHs as the main drug for VTE prophylaxis.While such effects of LMWH are known, it is not known whether they affect tendon healing.

In this study, we aimed to histopathologically compare the effects of LMWH, enoxaparin sodium treatment and HBO treatment, which are used to reduce the incidence of embolism prophylaxis due to immobilization after orthopedic surgeries, on Achilles tendon healing and to determine whether these treatments have positive or negative effects on healing.

## Methods

### Study design

In this study, a total of 36 male Wistar albino rats (age: three months [range, 2.5–3.5 months], weighing 200 g [range, 180–220 g]) (Duzce University Duzce Medical Faculty Experimental Animals Application and Research Center, Turkey) were used. Before the study, the necessary permission was obtained from the Ethics Committee (2021/02/03). The study was conducted in the Laboratory of the Experimental Animal Practice and Research Center of Duzce University. The principles of laboratory animal care were followed in this study. Our study was reported in accordance with ARRIVE guidelines and experimental animal ethics committee.

**Fig. 1:**
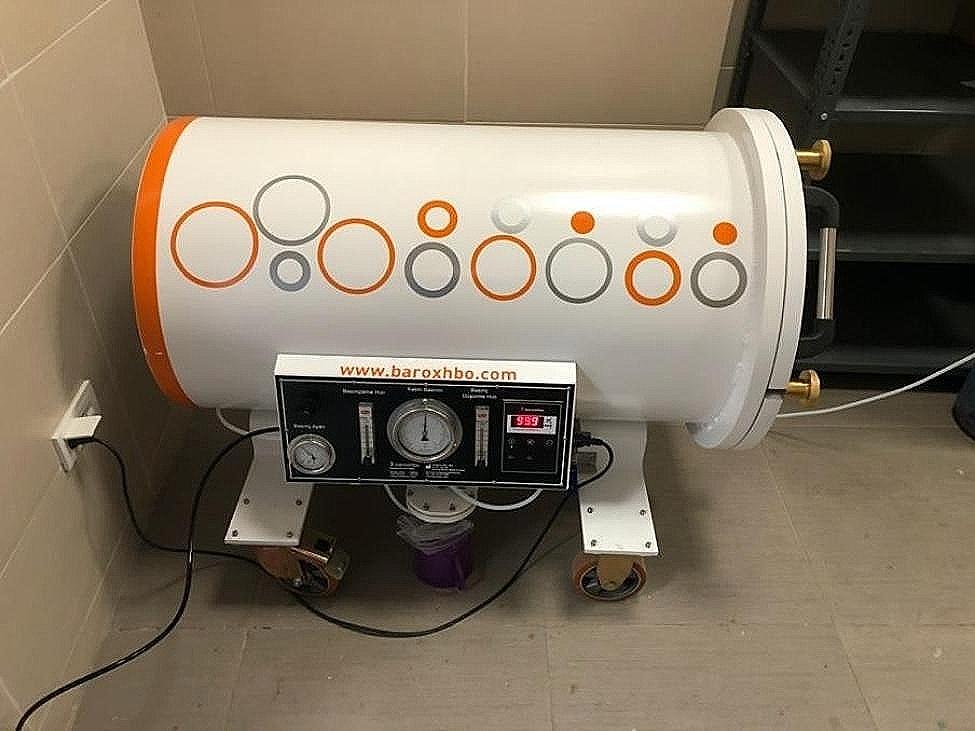
Hyperbaric oxygen chamber designed for animals

Animals were randomly divided into 4 groups with 9 rats in each cage and monitored in the laboratory for 1 week preoperatively. During the study period, the rats were given unlimited tap water (ad libitum) and standard rodent feed. The animals were caged in a room with a controlled temperature (23–25 °C) and a 12/12-hour light/dark cycle with 50–60% humidity. After the animals used in the study were brought to the laboratory center, they were followed in their cages for approximately 1 week without any treatment. On the day of the study, the rats were brought to the research laboratory and weighed. The HBO cabinet designed for animals is shown in Fig. 1.

### Surgical procedure

**Fig. 2:**
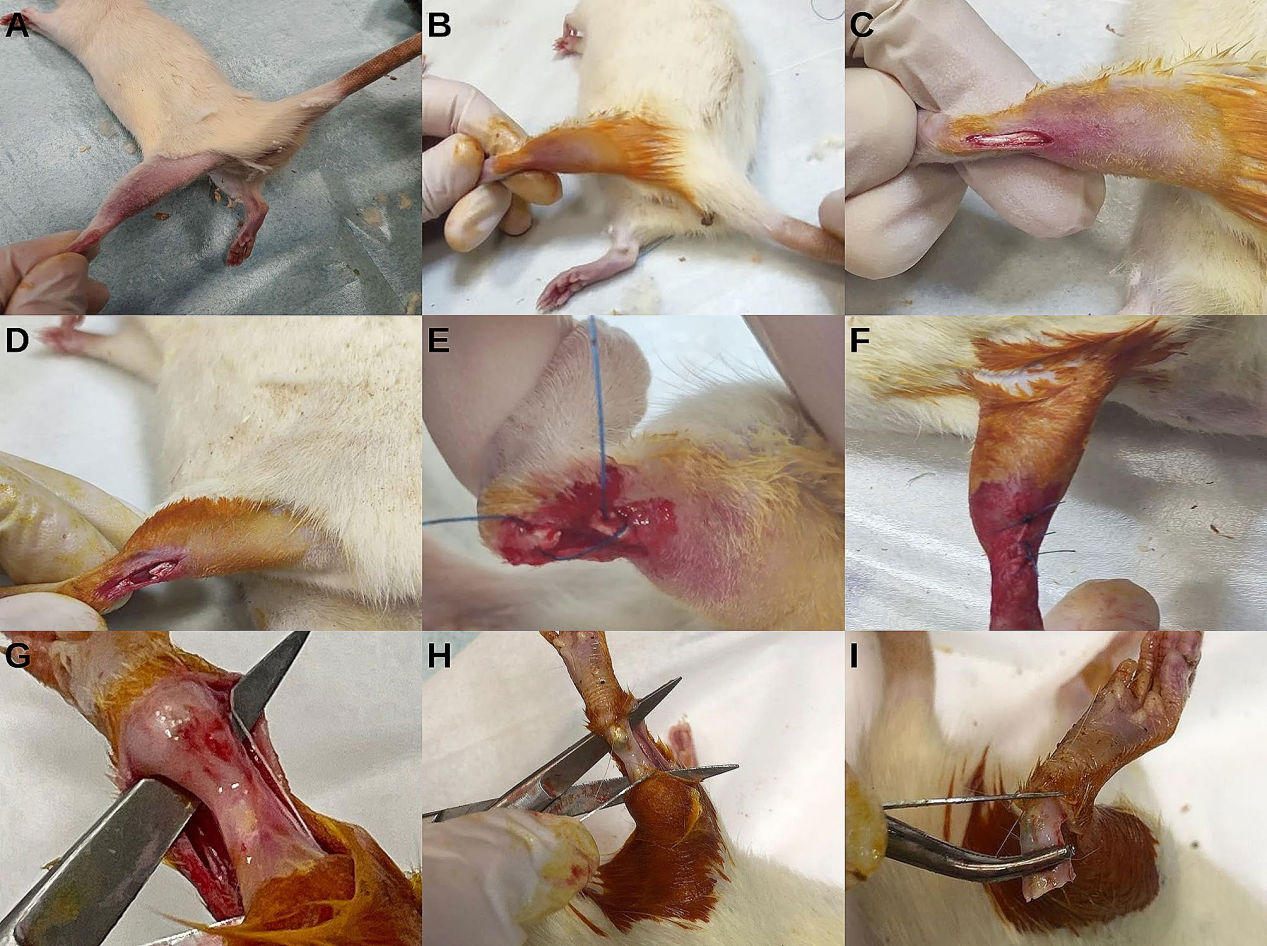
Surgical technique. ***A***: Preparation of the surgical field by shaving ***B***: Staining the surgical field with povidone iodide ***C***: Exposure of the Achilles tendon ***D***: Tenotomised Achilles tendon ***E***: End-to-end suturing of the tendon ***F***: Skin suturing and dressing ***G***: Accessing the tendon to be excised through the old incision ***H***: Incision made proximal to the tenotomy line ***I***: Incision made distal to the tenotomy line

The rats were anesthetized intramuscularly with ketamine (90 mg/kg) and xylazine (6 mg/kg), and the posterior regions of the left hindfoot were shaved, following the rules of asepsis and antisepsis. For the exposure of the Achilles tendon, a 2.5 cm long longitudinal midline incision was made over the tendon. The Achilles tendon was then cut horizontally 0.5 cm proximal to its attachment to the calcaneus with a No. 15 scalpel. No external fixation agent was applied to the rats, and they were allowed to move freely in the cage after surgery. Fentanyl citrate (0.02 mg/kg) was administered subcutaneously for analgesia for 3 days after surgery. The surgical technique used in experimental animals is shown in Fig. 2.

### Study groups

Prophylactic antibiotics were administered to all groups after surgery. No rats died in any group after the operation. No wound site infection was observed in any of the rats during follow-up. The control group (*n* = 9) was named Group 1, and the enoxaparin sodium group (*n* = 9) was named Group 2. The HBO group (*n* = 9) was used as Group 3, and the enoxaparin sodium and HBO groups (*n* = 9) were used as Group 4.

Treatment was started on the first postoperative day. A total of 21 sessions of HBO treatment were administered to 9 rats in Group 3 and Group 4 once a day. The treatment was performed in a 100 × 55 cm hyperbaric oxygen chamber specially built for experimental animals, where 18 rats could fit together with their cages at the same time. After 21 sessions, the treatment was terminated. The treatments were performed at 2.5 ATA pressure, and each session lasted 60 min in total. Before the treatment, the chamber was flushed with 100% oxygen for 5 min, after which the pressure in the chamber was gradually increased, and the desired treatment depth was reached within 5 min. Treatment was performed at 2.5 ATA pressure for 60 min, decompression was achieved in 10 min, and the treatment was terminated in a total of 80 min.

Eighteen rats were in Group 2, and 18 rats were in Group 4. enoxaparin sodium was administered subcutaneously at a single dose of 1 mg/kg/day in a standard manner. No complications were encountered.

### Histopathological examination

After excision of the Achilles tendons from the rats, the tissues were immersed in a 10% formalin fixative solution for three days. Following fixation, the tissues were rinsed overnight under running water. The next day, they were sequentially processed through a series of alcohol solutions (60%, 70%, 80%, and 100%) and then transferred to xylene. The tissues were embedded in paraffin, and 3 μm sections were obtained using a microtome. These sections were stored in containers and kept in an oven at 60 °C overnight for deparaffinization. Subsequently, routine hematoxylin and eosin (H&E) and Masson’s trichrome (MT) staining were performed for morphological assessment. The prepared slides were examined and captured under a microscope.

The tenocytes, collagen, ground substance, and vascularization were evaluated using the Bonar scoring system, which consists of four grades (0, 1, 2, and 3). A score of zero (0) indicates normal tissue structure, while a score of three (3) indicates abnormal appearance. Total scores ranged from 0 (normal tendon appearance, strong healing) to 12 (severe pathology, poor healing). Additionally, fiber structure, fiber arrangement, nuclear rounding, regional changes in cellularity, increased vascularization, decreased collagen staining, hyalinosis, and glycosaminoglycan (GAG) content were assessed using the Movin scoring system, which also consists of four grades (0, 1, 2, and 3). In both scoring systems, lower values indicate better healing, while higher values indicate poor healing. Scores of zero (0) represent normal tissue structure, and scores of three (3) represent abnormal appearance. Total scores ranged from 0 (normal tendon appearance, strong healing) to 24 (severe pathology, very poor healing) [13]. 

### Statistical analysis

The Kruskal‒Wallis test was used for group comparisons. For multiple comparisons, groups were compared with post hoc Bonferroni correction. The data are presented as medians, interquartile ranges and minimum-maximum values. Categorical variables were compared with the Fisher–Freeman–Halton test and are presented as numbers and percentages. All the statistical analyses were performed with the SPSS v.22 package, and a level of statistical significance was set at 0.05.

## Results

The groups were compared according to the results of statistical analysis based on the histopathological data. The weights of the rats were recorded before the surgical procedure and at regular intervals, and the weights were equal between the groups; no statistically significant difference was observed (*p* = 0.956). Histopathologic scoring between the groups is given in Table 1.

**Table 1:** Histopathological score distributions between groups

	Control	Enoxaparin sodium group	HBO therapy group	HBO and Enoxaparin sodium group	*p*
Acute inflammation					
0 1	7 (87.5%) 1 (12.5%)	7 (87.5%) 1 (12.5%)	7 (87.5%) 1 (12.5%)	8 (100%) 0 (0.0%)	1.000
Chronic inflammation					
0 1 2	4 (50.0%) 3 (37.5%) 1 (12.5%)	4 (50.0%) 3 (37.5%) 1 (12.5%)	5 (62.5%) 3 (37.5%) 0 (0.0%)	5 (62.5%) 3 (37.5%) 0 (0.0%)	1.000
Neovascularization					
0 1 2 3	0 (0.0%) 5 (62.5%) 3 (37.5%) 0 (0.0%)	0 (0.0%) 3 (37.5%) 4 (50.0%) 1 (12.5%)	2 (25.0%) 5 (62.5%) 1 (12.5%) 0 (0.0%)	0 (0.0%) 2 (25.0%) 1 (12.5%) 5 (62.5%)	0.004*
Proliferation					
1 2 3	0 (0.0%) 7 (87.5%) 1 (12.5%)	3 (37.5%) 5 (62.5%) 0 (0.0%)	2 (25.0%) 4 (50.0%) 2 (25.0%)	0 (0.0%) 0 (0.0%) 8 (100%)	< 0.001*
Fibrosis					
1 2 3	4 (50.0%) 4 (50.0%) 0 (0.0%)	0 (0.0%) 5 (62.5%) 3 (37.5%)	1 (12.5%) 6 (75.0%) 1 (12.5%)	5 (62.5%) 3 (37.5%) 0 (0.0%)	0.025*
Foreign object					
- +	2 (25.0%) 6 (75.0%)	5 (62.5%) 3 (37.5%)	6 (75.0%) 2 (25.0%)	3 (37.5%) 5 (62.5%)	0.233

*p < 0.05 HBO therapy: hyperbaric oxygen therapy

There was no significant difference between the groups in terms of acute inflammation (*p* = 0.785) or chronic inflammation (*p* = 0.827) scores, but there were significant differences in neovascularization (*p* = 0.009), proliferation (*p* < 0.001) and fibrosis (*p* = 0.006) scores. A statistical comparison of the histopathological findings between the groups is given in Table 2.

**Table 2:** Statistical comparison of histopathological findings between groups

	Control	Enoxaparin sodium group	HBO therapy group	HBO and Enoxaparin sodium group	*p*
Acute inflammation	0 (0) [0–1]	0 (0) [0–1]	0 (0) [0–1]	0 (0) [0–0]	0.785
Chronic inflammation	0.5 (1) [0–2]	0.5 (1) [0–2]	0 (1) [0–1]	0 (1) [0–1]	0.827
Neovascularization	1 (1) [1-2]	2 (1) [1-3]	1 (0.75) [0–2]	3 (1.75) [1-3]	0.009*
Proliferation	2 (0) [2-3]	2 (1) [1-2]	2 (1.5) [1-3]	3 (0) [3-3]	< 0.001*
Fibrosis	1.5 (1) [1-2]	2 (1) [2-3]	2 (0) [1-3]	1 (1) [1-2]	0.006*

*p < 0.05 for HBO therapy: Hyperbarıc Oxygen Therapy. Data are presented as the median (interquartile range) and [minimum-maximum] values.

**Fig. 3:**
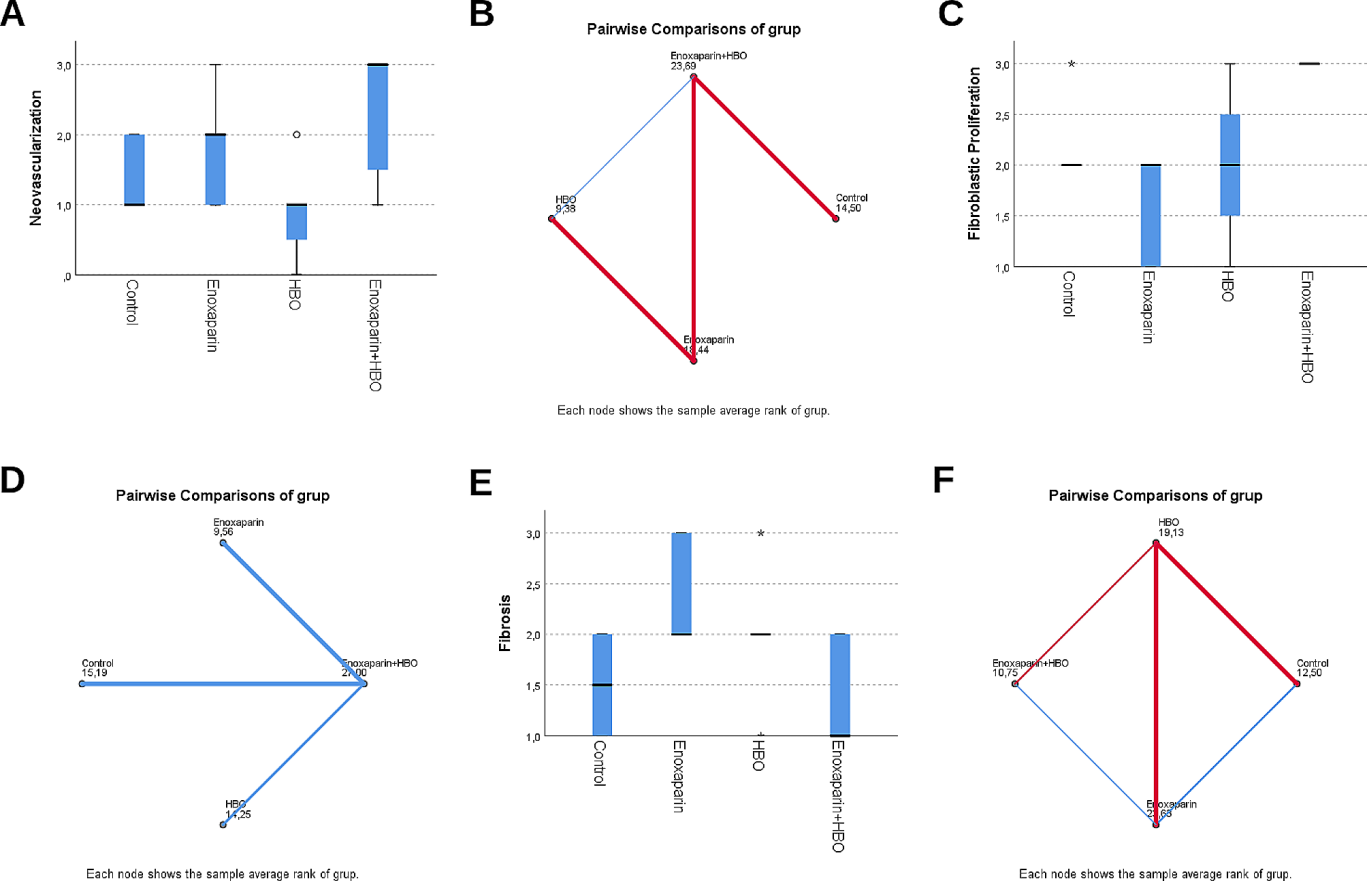
Box plots for fibrosis, fibroblastic proliferation and neovascularisation scores in groups. ***A***: Neovascularization levels in different treatments. ***B***: Pairwise comparisons of neovascularization groups. ***C***: Fibroblastic proliferation in different treatments. ***D***: Pairwise comparisons of fibroblastic proliferation. ***E***: Fibrosis levels in different treatments. ***F***: Pairwise comparisons of fibrosis groups

**Fig. 4:**
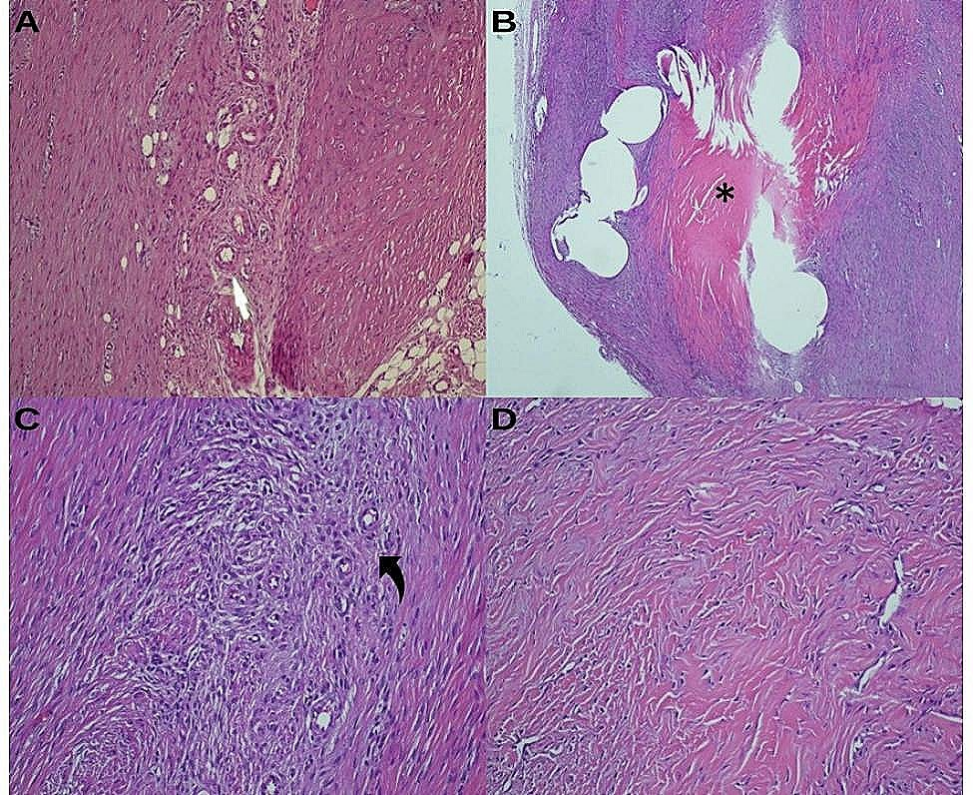
Histopathological microscope images. Light microscopy image showing increased vascularity (indicated with yellow arrow) in the enoxaparin group, H&E, X20 **(A)**, hyalinization area was indicated with asterisk in the hyperbaric therapy group, H&E, X10 **(B)**, round cell changes in the enoxaparin group (indicated with curvy arrow), H&E, X20 **(C)**, collagen alignment in the hyperbaric group was shown, H&E, X20 **(D)**

A significant difference in terms of neovascularization was observed only between the hyperbaric oxygen and enoxaparin sodium + HBO groups (*p* = 0.006); the other groups were similar according to pairwise comparisons. A significant difference in proliferation was observed in the enoxaparin sodium + HBO group compared with the other groups. The other groups were found to be similar when pairwise compared with the enoxaparin sodium and enoxaparin sodium + HBO (*p* < 0.001), hyperbaric oxygen and enoxaparin sodium + HBO (*p* = 0.017) and control and enoxaparin sodium + HBO (*p* = 0.034) groups. A significant difference in fibrosis was observed between the enoxaparin sodium group and the enoxaparin sodium + HBO group (*p* = 0.012) and between the control and enoxaparin sodium (*p* = 0.046) groups; the other groups were similar according to pairwise comparisons. Box plots for fibrosis, fibroblastic proliferation and neovascularisation scores and mean rank plots for multiple comparisons are given in Fig. 3. Histopathological images are also shown in Fig. 4.

## Discussion

In this study, we determined the effect of enoxaparin sodium, which is frequently used after orthopedic surgery, and HBO therapy, which has been used for musculoskeletal problems in the last year, on Achilles tendon healing in rats. Tendon healing is a complex process in which cell proliferation, angiogenesis, extracellular matrix production, remodeling and maturation follow each other, and some growth factors affect healing [14]. Fibrin formation is mediated by thrombin. In clinical practice, antithrombotic agents are usually given as a single daily dose to prevent continuous thrombin and fibrin formation [15, 16]. In some studies, antithrombotic agents were shown to positively affect healing by affecting the steps of the tendon healing process. However, it should be noted that these studies are very limited in number, and there are no studies in the literature investigating the effects of enoxaparin sodium on Achilles tendon healing [17, 18]. 

In some studies, acute postrupture findings, such as hemorrhage and inflammation, have been shown to be prominent in Achilles tendon specimens. The reflex system, which is normally activated when the muscle-tendon junction is overloaded and prevents stretching, is impaired, so muscle strength exceeds tendon strength and rupture occurs [19]. There is still no consensus about the treatment applied after Achilles tendon rupture [20]. However, whichever treatment method is chosen, the aim should be for the patient to return to social life quickly and to reach his/her former sportive level. According to studies performed in athletes, while the rupture rate is 10–30% with conservative treatment, this rate remains at 2–3% with appropriate surgical techniques. It has been reported that the risk of rupture, muscle atrophy, and stiffness in the related joints are lower and that the return to sports is faster with surgical treatment [21]. With conservative treatment, the risk of adhesions, deep vein thrombosis, muscle trophism and joint stiffness due to long-term immobilization is greater [22]. 

There are various studies on Achilles tendon healing in the literature. Some applications showing positive effects on Achilles tendon healing include laser, ultrasound, and direct current electrical applications [23]. Again, some studies have reported that injecting platelets into the rat Achilles tendon accelerates healing [24]. HBO has a positive effect on wound healing by increasing oxygen in tissues. Therefore, it increases the formation of new vessels in tissue with reduced vascularization [25]. In different studies, the effects of hyperbaric oxygen therapy on early tendon healing in the treatment of Achilles tendon ruptures have been investigated, and while conflicting results have emerged, additional studies are needed on this subject [26]. In some studies, similar to our study, HBO therapy had a positive histological and biomechanical effect on tendon healing after Achilles tendon repair [27]. 

In the literature, the effect of LMWH on Achilles tendon healing has been investigated in studies on rats, and similar positive effects were found in our study; however, compared with all the other groups, the HBO treatment significantly contributed to the tendon healing process [28–30]. 

Although positive effects of these HBO treatment methods on healing have been shown, a definite treatment protocol has not yet been established. According to the results of our study, we found histopathologically that HBO therapy accelerates healing after Achilles tendon repair. In addition, the pharmacologic agent enoxaparin sodium, which is mostly used for reducing DVT risk, has a positive effect on healing after Achilles tendon surgery. For this reason, orthopedic surgeons in particular may need to use the enoxaparin sodium group recommendation longer. However, studies in larger series are needed on this subject.

Since our study was an experimental study, no comparison could be made in terms of clinical or functional outcomes. Therefore, evaluating the results obtained in a manner similar to that used for Achilles tendon ruptures in humans may be misleading. Some of the important points that should not be overlooked when evaluating the results are the Achilles tendon incision we made, the age of the subject and the subject’s ability to heal. In humans, such injuries are usually seen as ruptures in middle-aged individuals, where degenerative changes occur and there is less vascularity. Our injury model was created by making a straight incision with a scalpel, and the rats were young. Although these conditions seem to constitute limitations of our study, because the same type of injury was created in the tissues of the damaged area, the subjects were the same age, the feeding and living conditions were the same, and the metabolism and healing potential of the animals were equal, thus allowing the effectiveness of the treatments to be reliably investigated. Care was taken to ensure that the number of subjects in the study was the minimum number of subjects that would be statistically significant within the framework of ethical rules by performing power analysis by examining previous studies.

The limitation of our study is that biomechanical evaluation could not be performed in our study. The reason why we could not perform this evaluation is due to the inadequacy of our laboratory conditions. One of the limiting factors in our study was histological evaluation. The lack of difference between the groups in histological examination may be due to giant cell reactions caused by the suture material passing through the tendon and the resulting separations. In addition, the lack of immunohistochemical analysis is another limitation of our study. As a result, the frequently observed artefacts may have adversely affected our histological evaluation. Another limiting factor is that the duration of tendon healing in rats is unknown [31]. According to the findings of our study, the use of enoxaparin sodium and hyperbaric oxygen had positive effects on the healing of Achilles tendon tears. Based on these results and considering the complications that may occur after Achilles tendon rupture, we believe that treatment with enoxaparin sodium and hyperbaric oxygen will be beneficial because it contributes to healing and prevents complications.

## Conclusion

This study demonstrated that both enoxaparin sodium and hyperbaric oxygen therapy (HBO) significantly enhance the healing of Achilles tendon ruptures in rats. We observed improvements in neovascularization, fibroblastic proliferation, and fibrosis in the treatment groups compared to the control group.Enoxaparin sodium, widely used for thromboprophylaxis, showed positive effects on tendon healing by stimulating macrophage activity and promoting new vessel formation. HBO therapy, known for its multiple therapeutic effects, significantly accelerated tissue healing. The combination of HBO and enoxaparin sodium yielded the most pronounced benefits, suggesting a synergistic effect. Enoxaparin sodium is primarily used to reduce the risk of thrombosis post-surgery and carries potential side effects such as bleeding. Therefore, while considering its beneficial effects on tendon healing, its primary purpose and potential risks must also be acknowledged. These findings suggest that the combined use of enoxaparin sodium and HBO therapy could be a potent therapeutic approach for improving Achilles tendon repair. Further research is needed to establish standardized protocols and assess long-term outcomes in clinical settings. In summary, the combined use of enoxaparin sodium and HBO therapy offers a promising strategy for enhancing Achilles tendon healing, potentially leading to quicker recovery and fewer complications after orthopedic surgeries.

## Data Availability

The datasets used and/or analyzed during the current study available from the corresponding author on reasonable request.
